# Encapsulation of Multiple Microalgal Cells via a Combination of Biomimetic Mineralization and LbL Coating

**DOI:** 10.3390/ma11020296

**Published:** 2018-02-13

**Authors:** Minjeong Kim, Myoung Gil Choi, Ho Won Ra, Seung Bin Park, Yong-Joo Kim, Kyubock Lee

**Affiliations:** 1 Department of Chemical and Biomolecular Engineering, Korea Advanced Institute of Science and Technology (KAIST), Daejeon 34141, Korea; kiminj218@gmail.com (M.K.); sbpark7@kaist.ac.kr (S.B.P.); 2 Graduate School of Energy Science and Technology, Chungnam National University, Daejeon 34134, Korea; cmg2465@gmail.com; 3 Clean Fuel Laboratory, Korea Institute of Energy Research, Daejeon 34129, Korea; Seojun@kier.re.kr; 4 Department of Biosystems Engineering, Chungnam National University, Daejeon 34129, Korea

**Keywords:** encapsulation, microalgae, microcapsule, mineralization, layer-by-layer, calcite

## Abstract

The encapsulation of living cells is appealing for its various applications to cell-based sensors, bioreactors, biocatalysts, and bioenergy. In this work, we introduce the encapsulation of multiple microalgal cells in hollow polymer shells of rhombohedral shape by the following sequential processes: embedding of microalgae in CaCO_3_ crystals; layer-by-layer (LbL) coating of polyelectrolytes; and removal of sacrificial crystals. The microcapsule size was controlled by the alteration of CaCO_3_ crystal size, which is dependent on CaCl_2_/Na_2_CO_3_ concentration. The microalgal cells could be embedded in CaCO_3_ crystals by a two-step process: heterogeneous nucleation of crystal on the cell surface followed by cell embedment by the subsequent growth of crystal. The surfaces of the microalgal cells were highly favorable for the crystal growth of calcite; thus, micrometer-sized microalgae could be perfectly occluded in the calcite crystal without changing its rhombohedral shape. The surfaces of the microcapsules, moreover, could be decorated with gold nanoparticles, Fe_3_O_4_ magnetic nanoparticles, and carbon nanotubes (CNTs), by which we would expect the functionalities of a light-triggered release, magnetic separation, and enhanced mechanical and electrical strength, respectively. This approach, entailing the encapsulation of microalgae in semi-permeable and hollow polymer microcapsules, has the potential for application to microbial-cell immobilization for high-biomass-concentration cultivation as well as various other bioapplications.

## 1. Introduction

Microcapsule synthesis has drawn considerable attention for its various promising applications in the fields of biotechnology, food processing, medicine, and pharmaceutics [1,2,3]. Microcapsules can be prepared with various materials via different methods, such as microfluidics [4], emulsion/phase separation techniques [5], nozzle reactor technology [6], and others. Living-cell encapsulation, which has a great many applications, such as cell-based sensors, bioreactors, and bioenergy, requires a careful selection of materials and fabrication techniques, since living cells are easily ruptured by even very weak mechanical and chemical stresses [7,8,9]. Living cells have been successfully encapsulated by a direct deposition of nanomaterials, shell mineralization on cell surfaces, or a layer-by-layer (LbL) coating of polyelectrolytes [10]. For the direct deposition of nanomaterials on cells, colloid particles such as nanoparticles, nanorods, nanotubes, and nanosheets can be used [11,12,13,14,15]. In order to induce interaction with cells, colloid particles have been grafted with chemical functional groups or adsorbed with molecules. In many cases, for better interaction with colloid particles, some initial cell-surface-modifying pretreatment should be performed. Inspired by nature’s several unicellular microorganisms with solid inorganic shells, such as diatoms and foraminifera, artificial biomimetic inorganic shells have been successfully mineralized on microbial cells [16,17]. Choi et al. encapsulated single cells of microalgae and yeast with cytocompatible TiO_2_ and SiO_2_ shells, respectively [9,18]. For the coating of polymeric layers on substrates, the LbL technique, which is based on the self-assembly of different alternating anionic and cationic polyelectrolytes, is widely utilized [19,20,21,22]. With this method, polymeric microcapsules of controlled size and permeability have been fabricated using micron-sized sacrificial templates for various applications, including the encapsulation of living microorganisms [23,24]. However, the above-mentioned methods are suitable only for the encapsulation of single cells. For the encapsulation of living cells within a biocompatible hydrogel matrix, extrusion, lithography, microfluidics, emulsion, and bioprinting have been applied [7]. By means of these methods, the number of encapsulated cells can be controlled by changing the concentration of suspended cells in a water-based solution containing a hydrogel precursor which subsequently undergoes gelation. The encapsulating materials, however, are restricted to mainly hydrogels. Furthermore, with these methods, the formation of hollow microcapsules is problematic. For the preparation of double-emulsion drops and semi-permeable hollow microcapsules, a capillary microfluidic device has been successfully utilized [4,25]. The main drawback of microfluidics, however, is poor scalability. The development of a new approach for the encapsulation of multiple living cells in hollow microcapsules, therefore, is required.

In the present study, we developed, for the encapsulation of multiple living microalgal cells, a novel strategy combining the LbL technique and biomimetic mineralization. Crystal growth of CaCO_3_ covering several microalgal cells resulted in the formation of microalgae-embedded CaCO_3_ crystals. Microalgae encapsulation subsequently could be achieved by coating the microalgae-embedded CaCO_3_ crystals with polyelectrolyte multilayers followed by the gentle removal of CaCO_3_ sacrificial templates. This approach provides the following advantages for the preparation of hollow microcapsules encasing multiple living cells. First, the size and shape of microcapsules, which are dependent on those of the CaCO_3_ sacrificial templates, can be controlled. Second, the confinement of living cells in the crystal can be a safe environment against hazardous and stressful conditions of polymer encapsulation. Third, the surface of microcapsules can be decorated with various functional nanomaterials, such as Au nanoparticles, Fe_3_O_4_ particles, or carbon nanotubes (CNTs). Fourth and lastly, the approach is up-scalable.

## 2. Materials and Methods

### 2.1. Synthesis of Cell-Embedded CaCO_3_ Crystals

**Figure 1:**
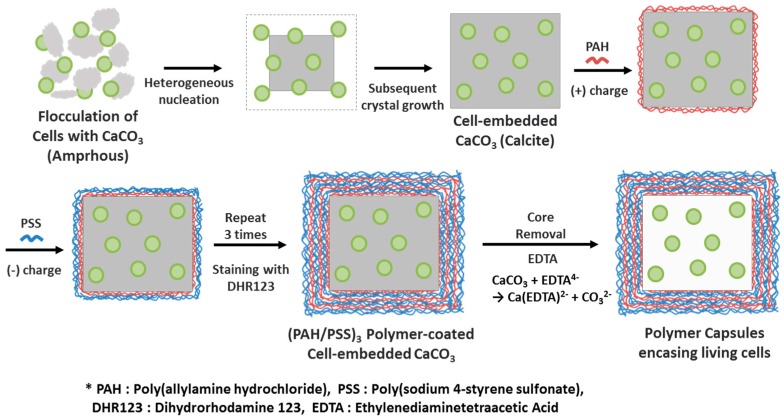
Illustrative scheme of the preparation of polymer capsules encasing living microalgal cells via CaCO_3_ mineralization and a layer-by-layer (LbL) polyelectrolyte coating followed by CaCO_3_ demineralization.

The microalgae (*Chlorella* sp. KR-1) were cultivated in one liter Pyrex bubble-column photobioreactors (0.5 L working volume) supplied with 10% (v/v) CO_2_ in air using a modified N8 medium [26]. The medium contained 3 mM KNO_3_, 5.44 mM KH_2_PO_4_, 1.83 mM Na_2_HPO_4_, 0.20 mM MgSO_4_·7H_2_O, 0.12 mM CaCl_2_, 0.03 mM FeNaEDTA, 0.01 mM ZnSO_4_·7H_2_O, 0.07 mM MnCl_2_·4H_2_O, 0.07 mM CuSO_4_, and 0.01 mM Al_2_(SO_4_)_3_·18H_2_O. The light intensity, temperature, and pH were maintained during cultivation at, respectively, about 80 µmol photons/m^2^∙s (using fluorescent lamps), 28–31 °C, and approximately 6.5. The microalgae used in the experiment were harvested after four to seven days of cultivation. Figure 1 shows a scheme for the encasing of microalgal cells in polymer microcapsules. As the first step, microalgal cells in culture medium solutions were centrifuged and washed with distilled water to remove culture medium. Cells were suspended in 1 mL of distilled water at a concentration of 1.4 mg∙cell/mL. The cell concentration was adjusted by the optical density at 660 nm. After 5 mL of 50 mM CaCl_2_ aqueous solution was mixed into 1 mL of cell-suspension solution, 5 mL of 50 mM Na_2_CO_3_ aqueous solution was added. After two hours, the precipitates were washed with distilled water to remove excess reactant. Then, the particles were treated by bath sonication for one minute to detach cells attached to the CaCO_3_ surfaces. Additionally, the concentration of CaCl_2_/Na_2_CO_3_ was varied to 10 and 100 mM while the other conditions were fixed in order to investigate the crystal size control by changing the concentration of CaCl_2_/Na_2_CO_3_.

### 2.2. Synthesis of Polyelectrolyte Capsules Encasing Several Living Cells

Cell-embedded CaCO_3_ crystals were introduced into poly(allylamine hydrochloride) (PAH, Mw 50 kDa, Sigma-Aldrich, Saint Louis, MO, USA) and poly(sodium 4-styrenesulfonate) (PSS, MW 70 kDa, Sigma-Aldrich) solutions (2 mg/mL, 0.5 M NaCl) sequentially with gentle shaking for 15 min. Depositions of PAH and PSS were each repeated three times, resulting in the adsorption of six layers onto the CaCO_3_ surface. For the final deposition, particles were stained with fluorescent dye dihydrorhodamine123 (DHR 123, Sigma-Aldrich) in PSS for 15 min for visualization of the capsules under fluorescent microscopy. After each adsorption, the particles were centrifuged and washed repeatedly at least three times with distilled water. The microcapsules also were functionalized with Au nanoparticles, Fe_3_O_4_ nanoparticles, and CNTs by charge neutralization. In these cases, the nanostructures were mixed with LbL-coated CaCO_3_ crystals after the fifth layer of coating with the PAH outermost layer. Au nanoparticles were purchased from Sigma-Aldrich (15 nm diameter, stabilized suspension in 0.1 mM PBS). Au nanoparticles were used as-received. Fe_3_O_4_ nanoparticles were prepared by a modified coprecipitation method. Briefly, 26 mmol of iron(III) chloride hexahydrate (FeCl_3_·6H_2_O, >98%, Sigma-Aldrich) and 13 mmol of iron(II) chloride tetrahydrate (FeCl_2_·4H_2_O, >99%, Sigma-Aldrich) were dissolved in 125 mL of distilled water. After thorough mixing, the solution was heated to 85 °C under a nitrogen environment for 30 min. Then, 8.4 mL of ammonium hydroxide solution (NH_4_OH, 25% NH_3_ in dH_2_O, Sigma-Aldrich) was slowly added to the mixture, which was maintained for 30 min. After the mixture was cooled to room temperature, Fe_3_O_4_ nanoparticles were washed with distilled water and ethanol by magnetic decantation. Fe_3_O_4_ nanoparticles were used as-prepared. The multi-walled CNTs (Ctube120, CNT Co., Ltd., Incheon, Korea) with average diameter of 20 nm, and length of 20–100 μm were used in this experiment. CNTs were dispersed in 1% sodium dodecyl sulfate (CH_3_(CH_2_)_11_OSO_3_Na, >98.5%, Sigma-Aldrich) aqueous solution followed by sonication for one hour and washing with distilled water by centrifugation. For microcapsule formation, the LbL-coated particles were introduced to a 0.2 M Ethylenediaminetetraacetic acid (EDTA, Sigma-Aldrich) solution of pH 7 for one hour to dissolve the CaCO_3_. The microcapsules were gently rinsed three times with distilled water and kept in N8 medium. For observation of cell growth in the microcapsules, a culture chamber holding 65 μL of solution was prepared using a Gene Frame^®^ (1.5 × 1.6 cm; Thermo Fisher Scientific Inc., Waltham, MA, USA) attached between a glass slide (bottom) and its cover glass (top). The glass slide with the culture chamber was maintained at 25 °C under 60% relative humidity for three days, and the cell growth was monitored by optical and fluorescent microscopy.

### 2.3. Characterization

For cell-concentration control purposes, the optical density was measured by UV-VIS spectrophotometry (Optizen 2120 UV, Mecasys Co., Daejeon, Korea). Zeta-potential measurements were performed in water using a Zetasizer (ZS90, Malvern, London, UK). Microalgal cells, CaCO_3_ crystals, polymer microcapsules, and their combinations were observed in the various bright-field, cross-polarization, and fluorescence modes of optical microscopy (Microscope Axio Imager A2, Carl Zeiss, Oberkochen, Germany). For the fluorescence images, a long-pass filter (Excitation filter: 450–490 nm band pass; BS FT: 510 nm; EM LP: 515 nm) was used. The number of cells embedded in the CaCO_3_ crystals was counted based on the depth-scanned images obtained by confocal laser scanning microscopy (C2plus, Nikon, Minato-ku, Japan). The number of cells per CaCO_3_ crystal was determined by counting and averaging the red autofluorescent signals from 17 randomly chosen crystals. Optical images were analyzed by Image J (Image J2, NIH, Bethesda, ML, USA) and AxioVision software (AxioVision LE, Carl Zeiss, Oberkochen, Germany). The morphology of the cell-embedded CaCO_3_ crystals and their sizes were investigated by field-emission scanning electron microscopy (FE-SEM; S-4800, Hitachi, Tokyo, Japan, and Nova 230, FEI, Hillsboro, OR, USA). The size of the crystals was determined by measuring 50 randomly chosen crystals and averaging. Transmission electron microscopy (TEM, Tecnai TF30 ST, FEI, Hillsboro, OR, USA) observation was carried out after focused ion beam (FIB) thin sectioning of microalgal-cell-embedded CaCO_3_ crystal by FIB-SEM (Helios Nanolab 450 F1, FEI, Hillsboro, OR, USA).

## 3. Results

### 3.1. CaCO_3_ Mineralization

**Figure 2:**
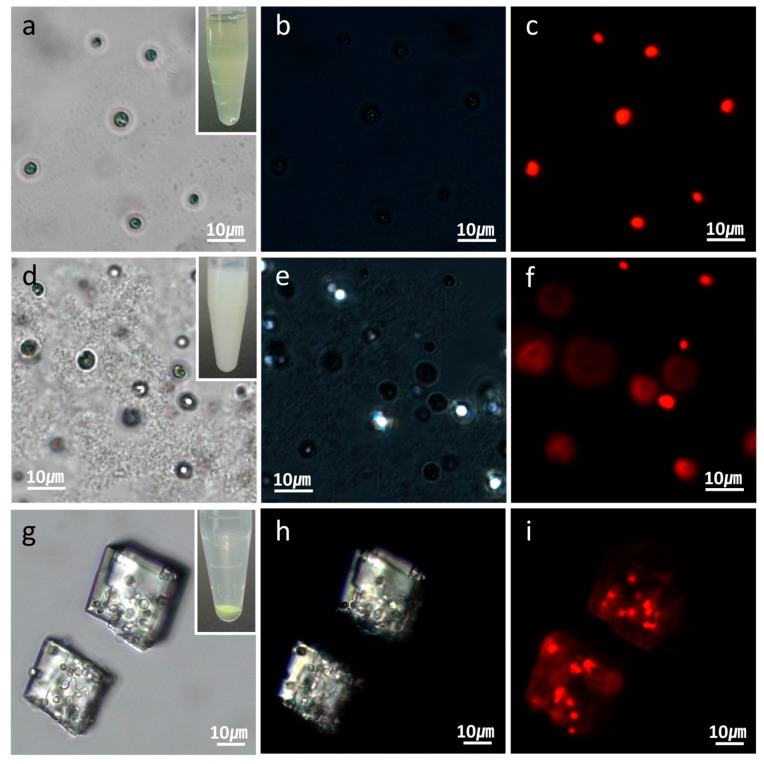
Optical-microscopic images of (**a**–**c**) microalgal suspension; (**d**–**f**) microalgal suspension immediately after the CaCl_2_ and Na_2_CO_3_ solutions were mixed; and (**g**–**i**) the same suspension with (**d**–**f**) after two hours. The optical-microscopic images are in the (**a**,**d**,**g**) bright-field; (**b**,**e**,**h**) cross-polarization; and (**c**,**f**,**i**) fluorescence modes. The red fluorescence in (**c**,**f**,**i**) originated from the autofluorescence of the microalgae’s chlorophyll.

**Figure 3:**
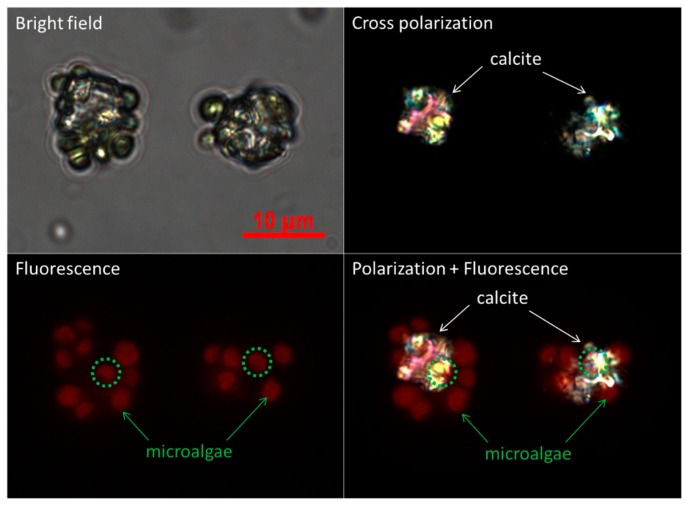
Optical microscopic images of the initial stage of CaCO_3_ crystals in the presence of microalgal cells in the mode of bright-field, cross-polarization, fluorescence, and the overlay of polarization and fluorescence.

Figure 1 illustrates the procedure for the preparation of polymer microcapsules encasing living microalgal cells. The process consists of three main steps: Embedding of microalgal cells in micron-sized CaCO_3_ crystals, LbL polyelectrolyte coating of cell-embedded CaCO_3_ crystals, and CaCO_3_ demineralization to form polymer microcapsules encasing microalgal cells. Figure 2 shows optical-microscopic images of a microalgal suspension (a–c), the microalgal suspension immediately after mixing of the CaCl_2_ and Na_2_CO_3_ solutions (d–f), and the same suspension with (d–f) after two hours (g–i) in the bright-field (a,d,g), cross-polarization (b,e,h), and fluorescence (c,f,i) modes, respectively. The red fluorescence originated from autofluorescence of the microalgae’s chlorophyll. When the CaCl_2_ and Na_2_CO_3_ solutions were mixed, white precipitates were observed immediately as shown in the inset of (Figure 2d). At this stage, the precipitates were mainly in the amorphous phase with only a few crystals, as revealed under cross-polarization microscopy (Figure 2e). After two hours, the greenish particles had completely settled, and the supernatant solution had become transparent (Figure 2g inset). Microscopic observation showed that several tens of micrometer-sized CaCO_3_ crystals of the typical rhombohedral shape of calcite had formed (Figure 2g,h). The red autofluorescence observed from the inside of the crystals established that several microalgae were entrapped therein (Figure 2i). The overlaid image of the cross-polarization and fluorescence microscope in Figure 3 shows clearly that at the initial stage, the CaCO_3_ crystals after heterogeneous nucleation are surrounded by multiple cells, which is illustrated in Figure 1 and will be explained in detail in the discussion section.

**Figure 4:**
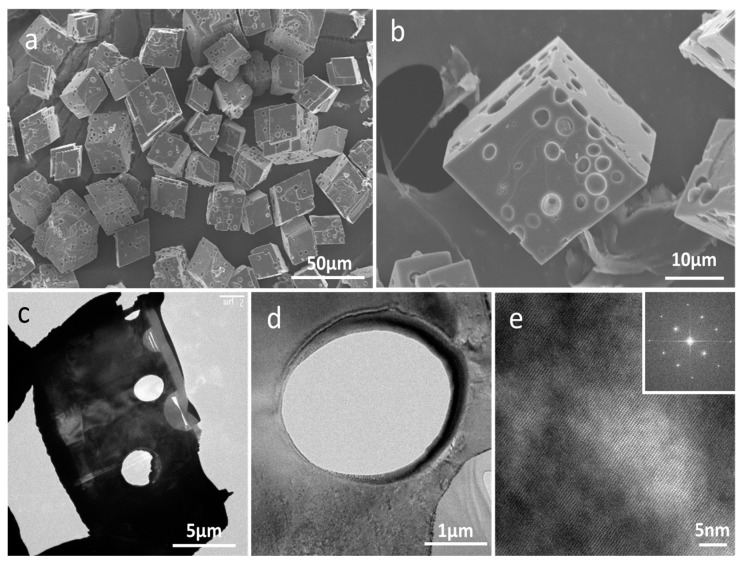
(**a**,**b**) SEM images of cell-embedded CaCO_3_ crystals; (**c**,**d**) TEM images of cell-embedded CaCO_3_ crystals after thin sectioning by focused ion beam (FIB); (**e**) HR-TEM image of (**d**) and (inset) the selected area electron diffraction (SAED) pattern.

SEM images showed the morphology of the cell-embedded CaCO_3_ crystals to be somewhat reminiscent of swiss cheese, with 2–3 μm holes in which microalgal cells were located (Figure 4a,b). Figure 4c,d provide TEM images of cell-embedded CaCO_3_ crystals after FIB thin sectioning. Round-shaped holes without facets are clearly apparent. The high-resolution TEM’s crystal lattice image and the selected area electron diffraction (SAED) pattern revealed the crystal to be in the single-crystalline phase (Figure 4e).

### 3.2. LbL Coating and CaCO_3_ Demineralization

**Figure 5:**
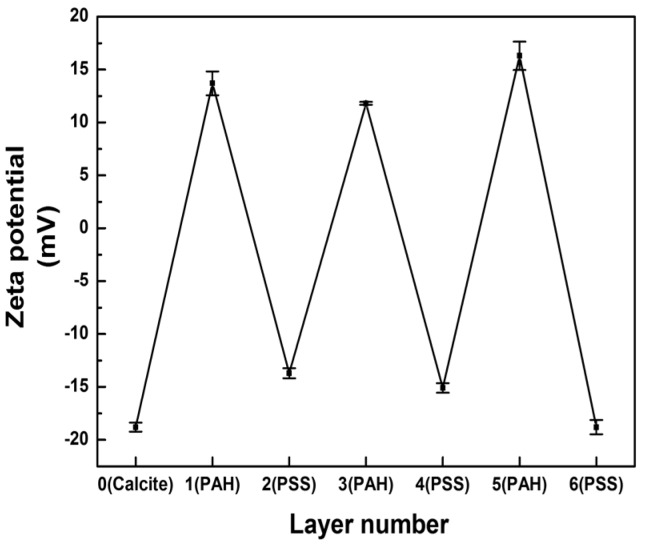
Zeta potentials of multilayers assembled by LbL coating of cell-embedded CaCO_3._

**Figure 6:**
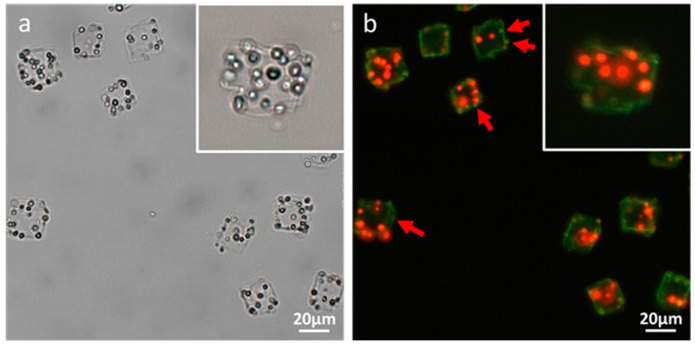
Optical-microscopic images of polymer microcapsules encasing microalgal cells in (**a**) bright-field and (**b**) fluorescence modes. The red and green fluorescences in (**b**) originated from the autofluorescence of the microalgae’s chlorophyll and the LbL coating’s DHR123, respectively.

Figure 5 plots the zeta potentials of cell-embedded CaCO_3_ crystals after LbL deposition of poly(allylamine hydrochloride) (PAH) and poly(sodium 4-styrenesulfonate) (PSS). The first PAH coating of the CaCO_3_ crystals resulted in a zeta-potential change from negative to positive. The subsequent deposition of the PSS layer changed the positive surface charge back to negative, which pattern was repeated until six layers had been deposited (PAH/PSS)_3_. The measured zeta-potential alternation between positive and negative values indicated that each layer of oppositely charged polyelectrolyte had been successfully coated onto the CaCO_3_ crystals. Then, the removal of the CaCO_3_ resulted in the formation of rectangular-shaped polymer microcapsules encasing several living microalgal cells, as observed under bright-field microscopy (Figure 6a). No single bright spot was observed under cross-polarization microscopy, which revealed that the CaCO_3_ crystals had not been encased in the microcapsules (image not shown). The fluorescence microscopy images, however, showed several red autofluorescent signals from the chlorophyll of microalgae encased by polyelectrolyte shells, themselves represented as green fluorescence from DHR123 (Figure 6b).

### 3.3. Control of Microapsule Size, Functionalization of Nanostructures, and Cell Growth

**Figure 7:**
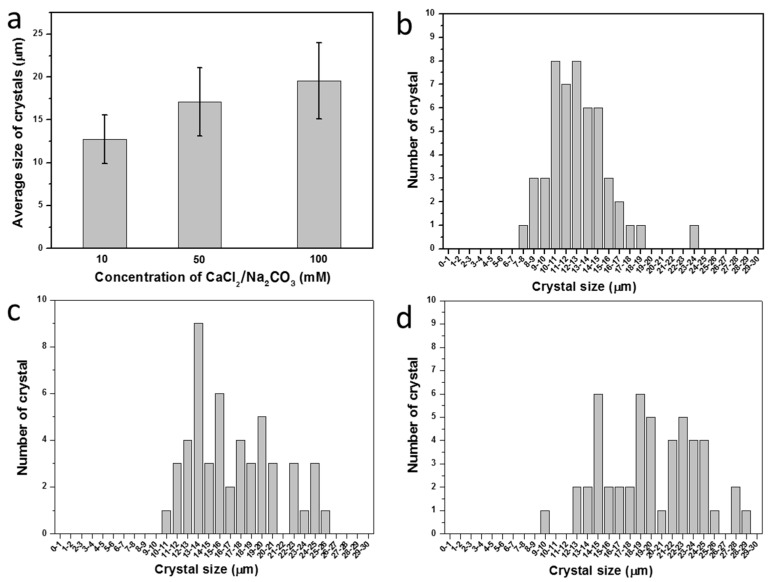
(**a**) Average sizes with standard deviations of CaCO_3_ crystals according to concentration of CaCl_2_/Na_2_CO_3_ precursor solution. The size distribution of CaCO_3_ crystals prepared at different concentrations of CaCl_2_/Na_2_CO_3_ precursor: (**b**) 10 mM; (**c**) 50 mM; and (**d**) 100 mM.

**Figure 8:**
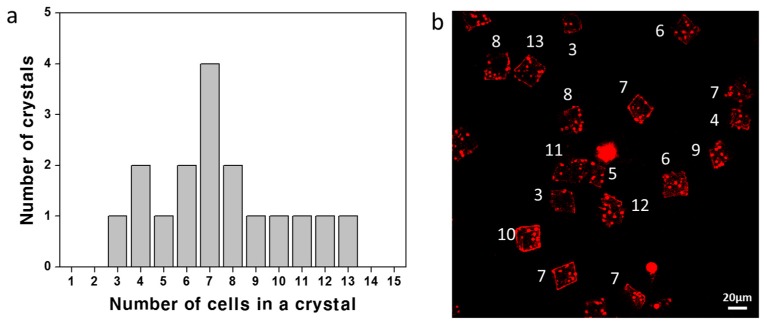
(**a**) The distribution of the cell number in a crystal (1.4 mg/mL cell and 50 mM CaCl_2_/Na_2_CO_3_) and (**b**) the corresponding confocal microscope image obtained by merging 15 depth-scanned images of red fluorescence (the white number indicating the cell counts). The average number of cells in a crystal is 7.47.

The size of CaCO_3_ crystals was controlled by alteration of the concentration of the CaCl_2_/Na_2_CO_3_ precursor solution. Figure 7a shows a plot of the average crystal size according to the concentration of CaCl_2_/Na_2_CO_3_. The size of a CaCO_3_ crystal increased as the concentration of CaCl_2_/Na_2_CO_3_ increased. The average sizes of the crystals at the precursor solution concentrations of 10 mM, 50 mM, and 100 mM were 12.7 ± 2.85 μm, 17.11 ± 3.95 μm, and 19.52 ± 4.43 μm, respectively. The size distributions of the crystals formed at each concentration are shown in Figure 7b–d with the corresponding SEM images (Figure S1). The distributions of the cell number per microcapsule at each concentration are also shown with the corresponding microscope images (Figure 8 and Figure S2).

**Figure 9:**
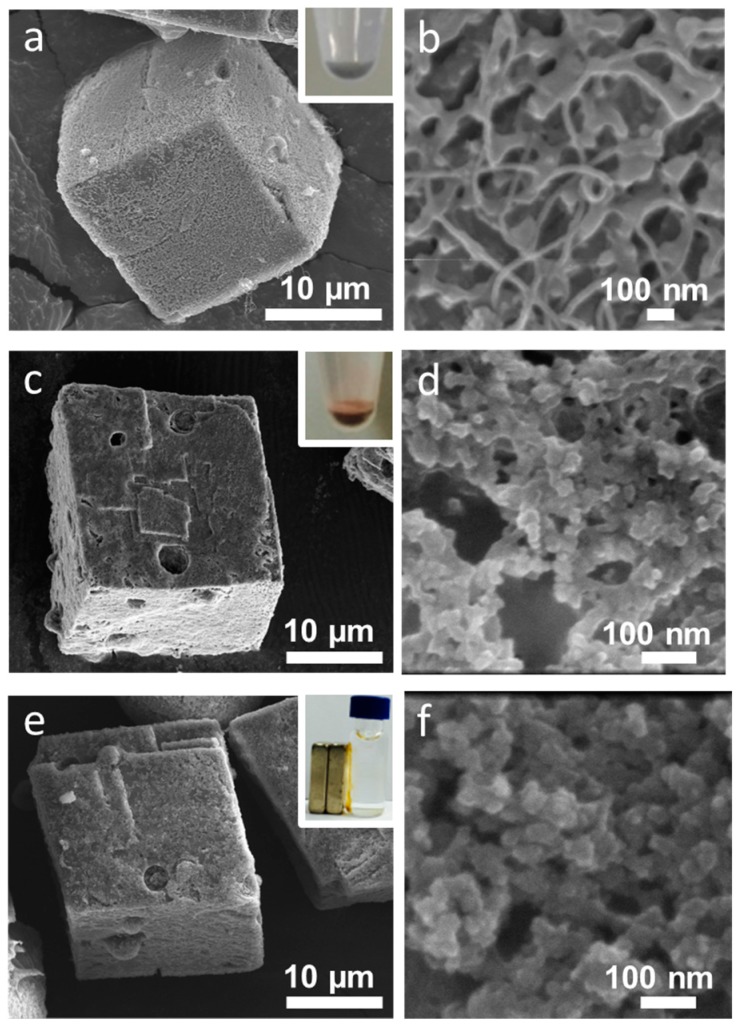
SEM images showing the functionalization of (**a**,**b**) carbon nanotubes (CNTs); (**c**,**d**) Au nanoparticles; and (**e**,**f**) Fe_3_O_4_ nanoparticles on polymer-coated CaCO_3_ crystals.

Polymer layers can be functionalized with various nanomaterials. Figure 9 shows SEM images of LbL-coated CaCO_3_ crystals functionalized with (a,b) CNTs, (c,d) Au nanoparticles, and (e,f) Fe_3_O_4_ nanoparticles. The images clearly show roughly textured surfaces coated with nanostructures. The negatively charged nanoparticles could easily be adsorbed onto the positively charged outer layer of the LbL coating by charge neutralization. The colored microcapsules shown in the inset of Figure 9 represent successful functionalization. The microcapsules functionalized with Fe_3_O_4_ nanoparticles could easily be separated by the application of a magnetic field.

**Figure 10:**
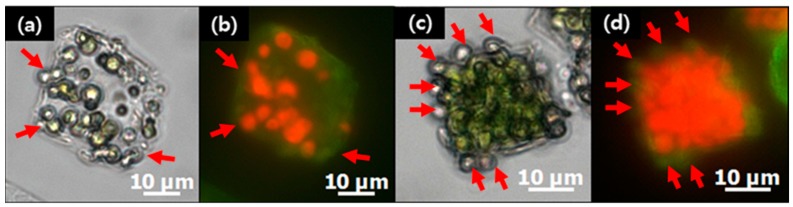
Optical-microscopic images of microalgal cells grown in the CaCO_3_ crystal-removed polymer microcapsule for (**a**,**b**) one day and (**c**,**d**) three days in the (**a**,**c**) bright-field and (**b**,**d**) fluorescent modes.

The cell growth in the microcapsules could be confirmed by microscopic observation in the bright-field and fluorescent modes. Figure 10 shows the proliferation of microalgae for one day (a,b) and three days (c,d). It is well-known that the polyelectrolyte shell formed by LbL coating has enough permeability for the diffusion of nutrients. These images serve to verify the permeability of the polyelectrolyte shell as well as the free space of the hollow microcapsules in which microalgae could proliferate.

## 4. Discussion

A mixture of CaCl_2_ and Na_2_CO_3_ solutions containing microalgae resulted in the formation of amorphous CaCO_3_ (ACC) aggregates in which microalgal cells were initially loosely entrapped. During the crystallization of ACC into calcite, microalgal cells became firmly fixed in the calcite matrix. It is notable that in spite of the embedding of round micrometer-sized microalgal cells, the typical rhombohedral shape of the single-crystalline calcite was not changed. Although many biological and synthetic examples of the occlusion of additives in single-crystalline calcite have been observed, the additives were mostly macromolecules or, at most, nanometer-sized particles [27,28,29,30]. Cho et al. recently visualized the propagation of step edges of calcite in the presence of micelles and demonstrated cavity formation and significant micelle compression during occlusion [31]. Han et al. also reported the formation of holes on the surface of the prismatic CaCO_3_, which were caused by the presence of cyanobacteria (*Synechocystis* sp.) [32]. They observed that the deformation of the holes originated from cells being squeezed by the constant pressure from the surrounding crystals of the hole as the small crystals grew. In our case, neither facet development nor clear cavities were observed in the TEM images of cell-embedded CaCO_3_ crystals after thin sectioning by FIB. The size and shape of the holes were exactly those of microalgae: 2–3 μm in diameter and round, respectively. Cho et al. showed that larger cavities were formed in the presence of larger micelles. The fact that cavities and facets are not formed during the occlusion of even micrometer-sized microalgae reveals that the surface of microalgal cells is highly favorable for the crystal growth of calcite. At the same time, the crystals are biocompatible for microalgal cells (*Chlorella* sp. *KR-1*) based on the fact that the cells survived and proliferated as shown in Figure 10 after being embedded in CaCO_3_ crystals for nearly two to three hours during crystallization and the LbL-coating process. It is known that, in general, microalgae have carboxylic (–COOH) and amine (–NH_2_) groups on their cell surfaces [33]; above pH 4–5, the carboxylic groups are negatively charged while the amine groups are uncharged, which results in a net negative surface charge. When the surface of microalgae was coated with PSS, however, microalgal cells were not successfully occluded in calcite. The optical-microscope images in Figure S3 show that cells with a negatively charged PSS outermost coating (PAH/PSS)_1_ are agglomerated and mostly attached on CaCO_3_ particles, which is a totally different pattern of crystallization from that in the presence of the bare cells. It clearly shows that the charge of the microalgal cell wall is not the sole parameter for the heterogeneous nucleation of CaCO_3_. We ascribe the highly efficient occlusion of microalgae in single-crystalline rhombohedral calcite to those unique functional groups on the cell surfaces.

A plausible mechanism to explain how microalgal cells are embedded in CaCO_3_ crystals is heterogeneous nucleation of crystal on the cell surface followed by cell embedment by the subsequent growth of crystal. The optical microscope images in Figure S4 reveal that most crystals contain microalgal cells. Red autofluorescent signals from microalgae are observed from all 20 crystals, as indicated by numbers, and only a few microalgal cells are excluded from crystals, as indicated by red arrows. This demonstrates that the microalgal cell wall constituents promote the heterogeneous nucleation of CaCO_3_ crystals. It is also supported by the previous study on the surface characterization of *Chlorella vulgaris*, which revealed that the presence of carboxyl groups on the cell surface was attributed to the adsorption of metallic cations by ion exchange [34]. In addition, as studied using various biological and synthetic models and reviewed by J.L. Arias and M.S. Fernández, polyanionic polysaccharides enhance the precipitation of calcium carbonate ions as well as the formation of calcite from nucleation to growth [35,36,37,38]. The optical microscopic images in Figure 3 show that at the initial stage, CaCO_3_ crystals after heterogeneous nucleation are surrounded by multiple cells. Based on the final crystal size and the number of cells embedded in the crystal, we can speculate that the cells are embedded in the crystals by subsequent crystal growth as illustrated in Figure 1.

Embedment of microalgal cells in crystals can provide the cells a safe environment against hazardous conditions, which is a crucial advantage of the current method of encapsulation. Especially, the direct contact of cell surfaces with the polymer coating and the precursor solutions of polyelectrolytes during the LbL coating process would be toxic to cells. In the fluorescent images of polymer-microcapsule-encased microalgal cells before and after proliferation, the red autofluorescent signals are not detected from the cells adsorbed on the surface, as indicated by red arrows (Figure 10b). This can be because the microalgal cells have lost their vitality, which is supported by the bright-field images showing the faded color of cells adsorbed on the surface (Figure 10a). The embedment of microalgal cells in CaCO_3_ crystals can play the roles not only of sacrificial templates but also of protecting cells from toxic environments. Other advantages of the mineralization–LbL encapsulation method is that the size of the capsule can be controlled as shown in Figure 7 and Figure S1. Furthermore, it is up-scalable. While methods such as extrusion, lithography, microfluidics, and bioprinting offer size-control advantages, the minimum size of the capsules produced is not smaller than 50 μm, and the scalability is not promising [7]. The emulsion method is up-scalable and can produce relatively small particles down to 10 μm; however, its main problem is that the microcapsule size distribution is quite wide and size control is not easy. In the mineralization–LbL encapsulation method, as shown in Figure 7, the size of CaCO_3_ crystals that determine that of the capsules can be controlled by changing the precursor concentration. The size of CaCO_3_ crystals can be even as small as several hundred nanometers. LbL coating of the surfaces of colloidal particles followed by core dissolution has been a widely used method to produce polymer capsules. CaCO_3_ is particularly attractive, with its ease of preparation and dissolution as well as biocompatibility for biological applications [19]. Furthermore, CaCO_3_ core particles can be synthesized to various shapes according to the crystalline phase and by using natural and synthetic additives. Yashchenok et al. synthesized anisotropic polyelectrolyte microcapsules of spherical, elliptical, and square shapes [39]. They adjusted synthetic parameters, such as mixing speed, time, and pH value to control the morphology of the core CaCO_3_ sacrificial particles. More complicated morphological control of CaCO_3_ is possible by using additives such as simple inorganic ions, low-molecular-mass organic additives, double-hydrophilic block copolymers, polar peptides, and proteins [40].

For various bioapplications, the modification of microcapsule surfaces with functional nanomaterials is possible, which is another advantage of the LbL encapsulation method. Different types of charged nanostructures can be attached to the surfaces of microcapsules by electrostatic interactions between the nanostructures and the oppositely charged polyelectrolyte layer. Microcapsules functionalized with magnetic nanoparticles can be moved in a desired direction remotely by an application of an external magnetic field [41]. Au-nanoparticle-functionalized microcapsules can be remotely damaged by infrared (IR) light illumination, resulting in the release of encapsulated materials [42]. Microcapsules assembled with two-dimensional (2D) nanostructures, such as CNTs, can be endowed with improved mechanical and electrical properties without sacrificing permeability at the molecular level [43].

Microalgae have received wide attention as one of the most promising sources of high-value biochemicals, such as β-carotene, astaxanthin, docosahexaenoic acid, and phycobilin pigments in the academic and industrial fields of cosmaceuticals, nutraceuticals, and functional foods [44]. Microalgae are grown either in a closed photobioreactor (PBR) or in open outdoor ponds. A PBR, often limited to microalgal culturing for high-value biochemicals, faces, as its main problem, high costs of construction and operation; therefore, much effort has been expended in order to obtain high biomass concentrations. To that end, the immobilization of microbial cells, with its ease of cell separation and low contamination risk, is a widely employed method. Optically transparent silica beads and biocompatible natural polymers, such as alginate or agarose, have been shown to be effective for immobilized culture applications [7,45,46,47]. While calcium alginate, with its simple synthetic procedure [48], is the most commonly used natural polymer for the microencapsulation of cells, thermal stability and swelling are issues of concern. Encapsulating microalgae in semi-permeable and hollow polymer microcapsules, as shown in Figure 10, is a promising option for the successful cultivation of high-biomass-concentration microalgae.

## Supplementary Materials

The following are available online at http://www.mdpi.com/1996-1944/11/2/296/s1, Figure S1: SEM images of the CaCO_3_ crystals formed at each concentration, Figure S2: The confocal microscope images scanned at different depth of cell-embedded CaCO_3_ crystals (1.4 mg/mL cell and 50 mM CaCl_2_/Na_2_CO_3_), Figure S3: Optical microscope images of CaCO_3_ crystals formed in the presence of microalgal cells with a negatively charged PSS outermost coating (PAH/PSS)_1_, Figure S4: Optical microscope images showing that most crystals contain microalgal cells.
