# Supplementary material for: Encapsulation of Multiple Microalgal Cells via a Combination of Biomimetic Mineralization and LbL Coating
Source: Materials (Basel). 2018 Feb 13;11(2):296. doi: 10.3390/ma11020296 (PMC5848993; doi:10.3390/ma11020296)
Supplement: Supplementary file 1 [file materials-11-00296-s001.pdf]

Supplementary Materials

Article

Encapsulation of Multiple Microalgal Cells via a Combination of Biomimetic Mineralization and LbL Coating

Minjeong Kim [1,†,‡], Myoung Gil Choi [2,†], Ho Won Ra [3], Seung Bin Park [1], Yong-Joo Kim [4,*], and Kyubock Lee [2,*]

[1] Department of Chemical and Biomolecular Engineering, Korea Advanced Institute of Science and Technology (KAIST), Daejeon 34141, Korea; kiminj218@gmail.com (M.K.); sbpark7@kaist.ac.kr (S.B.P.)

[2] Graduate School of Energy Science and Technology, Chungnam National University, Daejeon 34134, Korea; cmg2465@gmail.com

[3] Clean Fuel Laboratory, Korea Institute of Energy Research, Daejeon 34129, Korea; Seojun@kier.re.kr

[4] Department of Biosystems Engineering, Chungnam National University, Daejeon 34129, Korea

* Correspondence: babina@cnu.ac.kr (Y.-J.K.); kyubock.lee@cnu.ac.kr (K.L.); Tel.: +82-42-821-8610 (K.L.)

† These authors contributed equally to this work.

‡ Current Address: Amorepacific R&D Center, Korea.

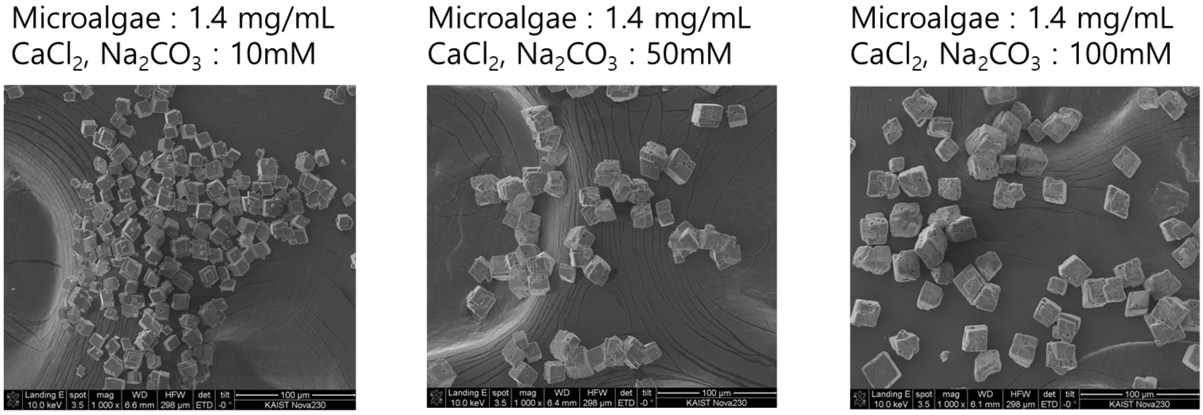

Figure S1. SEM images of the $CaCO_3$ crystals formed at each concentration.

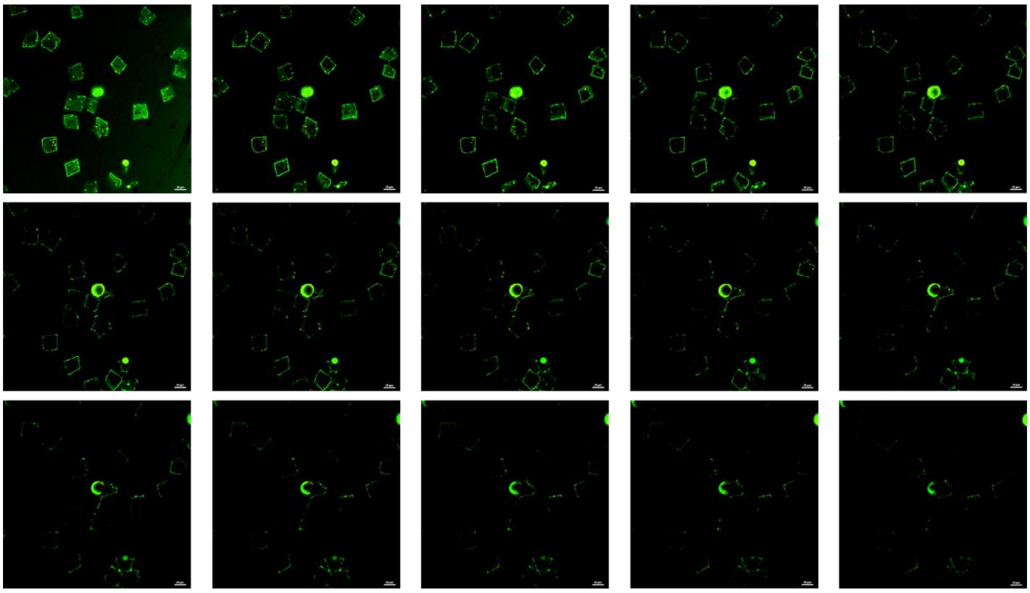

Figure S2. The confocal microscope images scanned at different depths of cell-embedded $CaCO_3$ crystals (1.4 mg/mL cell and 50 mM $CaCl_2$/Na_2CO_3)

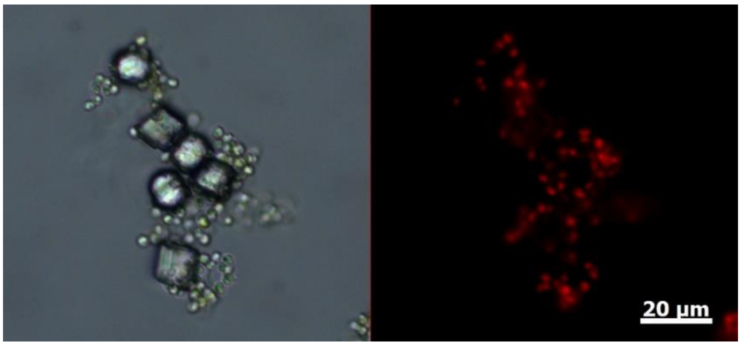

Figure S3. Optical microscope images of $CaCO_3$ crystals formed in the presence of microalgal cells with a negatively charged PSS outermost coating $(PAH/PSS)_1$. The images show that cells are agglomerated and mostly attached on $CaCO_3$ particles, which is a totally different pattern of crystallization from that in the presence of the bare cells.

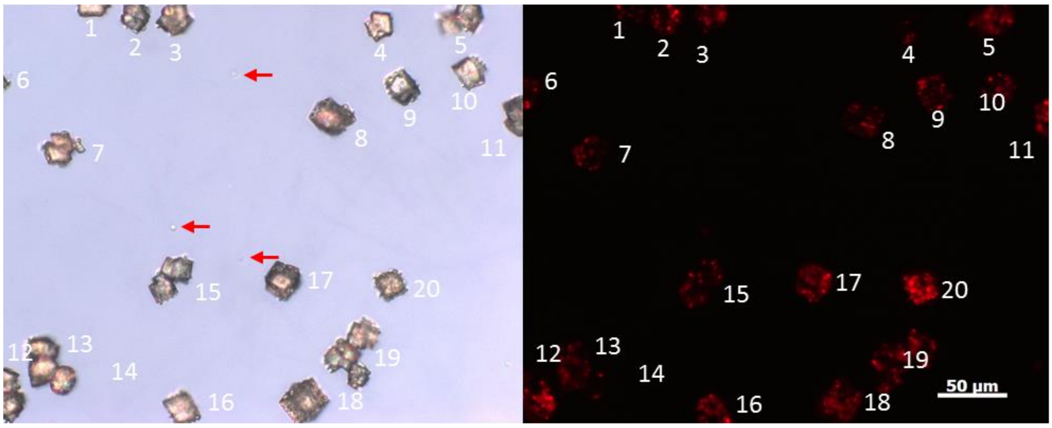

Figure S4. Optical microscope images showing that most crystals contain microalgal cells. Red autofluorescent signals from microalgae are observed from all 20 crystals, as indicated by numbers, and only a few microalgal cells are excluded from crystals, as indicated by red arrows.
